# Évaluation du modèle de gouvernance de la couverture médicale de base au Maroc

**DOI:** 10.11604/pamj.2021.38.210.25351

**Published:** 2021-02-24

**Authors:** Karsi Mohammed, Bennana Ahmed

**Affiliations:** 1 Centre d’Etudes Doctorales Sciences de la Vie et de la Santé, Université Mohammed V Souissi, Faculté de Médecine et de Pharmacie, Rabat, Maroc

**Keywords:** Evaluation qualitative, gouvernance, assurance maladie, sécurité sociale, Qualitative assessment, governance, health insurance, social security

## Abstract

**Introduction:**

cette étude évalue le modèle de la gouvernance de la couverture médicale de base au Maroc. L´objectif principal est de trouver un référentiel d´évaluation et l´appliquer sur le modèle marocain afin de proposer des recommandations d´amélioration.

**Méthodes:**

la méthodologie se repose sur l´analyse documentaire et des entretiens avec les représentants des parties prenantes sur la base d´un référentiel relatif à l´évaluation de la gouvernance des systèmes de la couverture médicale de base (CMB).

**Résultats:**

le modèle de gouvernance de la CMB a connu plusieurs changements depuis le lancement de la loi 65-00, l´évaluation a montré qu´il y a un effort considérable pour les dimensions: intervention des parties prenantes et la cohérence et la stabilité du système, cependant, les dimensions: supervision et régulation, cohérence de la structure de prise de décision et transparence et accès à l´information contiennent des problèmes structurants qui nuisent au bon fonctionnement du système.

**Conclusion:**

le modèle de gouvernance de la CMB au Maroc, nécessite une restructuration et une convergence des caisses de gestion pour un seul organisme qui centralise la gestion. Un effort à fournir aussi pour le volet régulation via l´Agence Nationale de l´Assurance Maladie qui a montré ses limites.

## Abstract (en)

**Introduction:**

the purpose of this study was to evaluate the governance model in basic health insurance coverage in Morocco. The main objective is to identify an evaluation framework and to apply it in the Moroccan model in order to propose recommendations for improvement.

**Methods:**

we conducted a literature review and interviews with representative stakeholders on the basis of a benchmark for evaluation of the governance of basic healthcare coverage (BHC).

**Results:**

the governance framework of BHC has undergone several changes since the launch of Law 65-00. Our evaluation has shown that considerable effort is being made given the dimensions: stakeholder involvement and system consistency and stability. However, dimensions such as supervision and regulation, coherence in decision-making structure, and transparency and access to information have structural issues that affect the proper functioning of the system.

**Conclusion:**

the governance framework of basic healthcare coverage in Morocco requires restructuring and convergence of the management funds for a single organization that centralizes the management. An effort should also be made to implement a regulation coordinated by the National Health Insurance Agency which has shown its limits.

## Introduction

La couverture médicale de base est le système que l´Etat met en place pour assurer une protection du citoyen contre le risque maladie comme les autres risques sociaux liés à la sécurité sociale (vieillissement, accident de travail etc...). La couverture du risque maladie, comme étant un service de l´Etat, est instaurée pour deux raisons: supporter le système de protection sociale qui couvre les autres risques sociaux et améliorer l´accessibilité financière du citoyen à l´offre de soins en participant au financement du système de santé.

Cette double mission, existait depuis la création des premiers modèles de l´assurance maladie obligatoire. La CMB a une double appartenance ou tutelle comme un service de l´Etat. D´un côté, elle contient des composantes favorisant son association dans la gestion avec les autres risques sociaux dans un programme de protection sociale gouvernemental global. De l´autre côté, sa contribution dans le financement des dépenses de santé et son rôle important dans la maitrise des ressources financières, rendent plus rationnel l´intégration de sa gestion dans une vision globale du système de santé. D´où vient la pertinence de s´intéresser à l´évaluation de la gouvernance de ce chantier avec cette interconnexion entre plusieurs intervenants. Il y a plus que 15 ans, l´idée selon laquelle la plupart des nations du monde s´engageraient à mettre en place une couverture sanitaire universelle (CSU) était considérée comme improbable et certainement pas comme une priorité pour la communauté de la santé mondiale. Nous avons passé de l´assurance maladie obligatoire à une nouvelle vision de la couverture du risque maladie [1].

Le but de la couverture universelle en matière de santé est de faire en sorte que tous les individus aient accès aux services de santé dont ils ont besoin sans que cela n´entraîne pour les usagers de difficultés financières [1]. La CSU repose sur trois composantes inter-associées: un éventail complet de services de santé en fonction des besoins, une protection financière contre le paiement direct des services de santé utilisés et la couverture de l´ensemble de la population. En ce sens, nous pouvons nous interroger sur l´impact que peut apporter cette nouvelle vision sur l´approche des décideurs pour améliorer les modèles de gouvernance de la CMB mis en place. Le Maroc à son tour est tenu de s´aligner avec les orientations des organismes internationaux et appliquer les résolutions de l´Organisation Mondiale de la Santé (OMS) en 2005 et 2011 (WHA58.33 et WHA64.9) [2] liées à la couverture médicale universelle.

Le modèle marocain de gouvernance de la CMB a une spécificité particulière. Il est un système mixte composé de deux pôles: l´assurance maladie obligatoire (AMO) qui se base sur l´assurance maladie (bismarckien), et le régime d´assistance médicale (RAMED) qui se base sur le système national (beveridgien). Dans notre travail, nous allons nous focaliser sur la gouvernance de ce chantier en déterminant ses composantes pour pouvoir analyser le modèle actuel. Force est de constater également que la gouvernance en tant que sujet de recherche portant ambiguïté dans sa définition, notre travail consiste à éclaircir le champ d´intervention de la gouvernance pour un chantier public tel que la CMB en se basant sur l´exemple marocain. Face à l´ambiguïté autour du mot gouvernance et la spécificité du chantier de la couverture médicale, comment peut-on procéder à l´évaluation du modèle de gouvernance de la CMB au Maroc?

## Méthodes

En premier lieu, nous avons procédé à une recherche dans le moteur de recherche « Google » (mots clés en annexe) et sur les sites web des organismes (répertoriés dans les références) sur toutes les publications officielles et institutionnelles sur la gouvernance de la CMB au Maroc. Les critères d´inclusion que nous avons fixés sont: articles ou rapports qui décrit un référentiel d´évaluation des systèmes de gouvernance de la CMB à l´échelle internationale; rapport décrivant la gouvernance du système de CMB au Maroc. Les critères d´exclusion: articles traitant la gouvernance de la CMB de manière générique; rapports institutionnels décrivant le système de gouvernance de la CMB avant la sortie de la loi 65-00 en 2002.

En deuxième lieu, nous avons procédé à une série d´entretien sur la base d´un guide semi-directif (en annexe) avec sept représentants des acteurs du système de la CMB au Maroc à savoir: la chefferie du gouvernement, l´agence nationale de l´assurance maladie (ANAM); la caisse nationale de sécurité sociale (CNSS); la caisse nationale des organismes de prévoyance sociale (CNOPS); l´autorité de contrôle des assurances et de la prévoyance sociale (ACAPS); le ministère de la santé; le ministère du travail et de l'insertion professionnelle. Il est à noter que nous avons trouvé une difficulté pour sélectionner les interlocuteurs des organismes qui peuvent avoir une vision globale sur la gouvernance de la CMB qui est un sujet discuté souvent dans le management stratégique. En plus, nous n´avons pas pu interroger un représentant du Ministère de l´intérieur durant la période réalisation de notre travail. Les entretiens se sont déroulés en respectant le principe de l´anonymat afin d´ajouter des commentaires à notre analyse documentaire.

En troisième lieu, nous avons choisi un référentiel d´évaluation des modèles de la gouvernance de la CMB, élaboré par la banque mondiale en 2008, qu´on va appliquer sur le modèle marocain. Le modèle présente les éléments de la bonne gouvernance qui s'appliquent à cinq dimensions importantes [3]: cohérence de la structure de prise de décision (Tableau 1); intervention des parties prenantes (Tableau 2); transparence et l´accès à l'information (Tableau 3); supervision et régulation (Tableau 4); cohérence et stabilité (Tableau 5). Chaque dimension est expliquée par des fonctions. Ces fonctions ne sont pas conçues comme des mesures unidimensionnelles du progrès vers la bonne gouvernance. En effet, chaque fonction interagit avec les composantes du système de la CMB lui-même. Nous allons présenter cette section en commençant par une description du système de gouvernance de la CMB au Maroc avant de présenter les résultats selon les méthodes utilisées: l´analyse documentaire, les entretiens et les dimensions et les fonctions du référentiel de la banque mondiale. Cela va nous permettre de mieux structurer les informations collectées.

**Tableau 1:** dimension cohérence de la structure de la prise de décision

Fonction	Indicateurs
1.La responsabilité des objectifs de la CMB doit correspondre au pouvoir et à la capacité de décision dans chaque institution impliquée dans la gestion du système	Oui /Non Exemples: L´institution responsable de la viabilité financière du système doit être capable de changer au moins l´un des paramètres dont il dépend (par ex. conditions d´affiliation, taux de cotisation, panier de soins, capacité d´agir comme acheteur stratégique, ou tarifs). L´institution chargée de la surveillance des caisses de la maladie ont la capacité de s´acquitter de ses responsabilités (c´est-à-dire qu´il a suffisamment de personnel qualifié, il a accès aux informations nécessaires et les textes juridiques lui donnent l´autorité pour remplir son rôle vis-à-vis des fonds de maladie).
2.Tous les organismes de la CMB procèdent régulièrement à l´évaluation du risque et stratégies de gestion mises en place	Oui /Non Exemple: Régulation claire dans les organismes de la CMB: l´évaluation continue du risque et la gestion du risque sont en place. Des stratégies sont en place, c´est-à-dire les organismes de la CMB suivent et analysent l´évolution des dépenses et contributions. Les organismes de la CMB ont la capacité de gérer les risques, c´est-à-dire prendre des mesures correctives afin d´assurer la viabilité financière du système en modifiant certains paramètres qui l´influencent (contribution taux de remboursement, composition dès l´ensemble des prestations, etc.).
3.Le coût de la régulation et l´administration des institutions de la CMB sont raisonnables et appropriés.	Oui/ Non Exemple: Les coûts maximum d´administration pour les organismes de la CMB sont mentionnés dans des textes juridiques ou dans les outils de régulation. Les coûts administratifs sont surveillés par le régulateur. Les provisions pour couvrir les coûts du régulateur de la CMB sont mentionnées dans les textes juridiques. Avant la mise en place d´une nouvelle mesure de régulation, une évaluation du coût des prestations est réalisée.

Source: Savedoff WD. Governing mandatory health insurance: concepts, framework, and cases. Gov Mandat Health Insur Learn Exp Wash DC World Bank. 2008;13-48. (Traduction libre)

**Tableau 2:** dimension intervention des parties prenantes

Fonction	Indicateurs
4.Les parties prenantes sont effectivement représentées dans organes de gouvernance des entités de la CMB	Oui/ Non Exemples: Organes de gouvernance de surveillance réglementaire et la gouvernance institutionnelle (conseil d´administration, organe de surveillance) ont des représentants des organismes gouvernementaux, organismes de réglementation, entités de la CMB, syndicats, organisations d´employeurs, les bénéficiaires, les fournisseurs et les experts indépendants. La représentation est efficace, à savoir les différentes parties prenantes. les points de vue sont pris en compte dans la prise de décision.

Source: Savedoff WD. Governing mandatory health insurance: concepts, framework, and cases. Gov Mandat Health Insur Learn Exp Wash DC World Bank. 2008;13-48. (Traduction libre)

**Tableau 3:** dimension transparence et accès à l´information

Fonction	Indicateurs
5.Les objectifs de la CMB sont formalisés et définis clairement	Oui /Non Exemples: Les objectifs sont énoncés dans un texte juridique de haut niveau (la Constitution ou une loi). Les objectifs sont publiés et facilement accessibles au public. Les objectifs sont clairement définis et facilement compréhensible.
6.La CMB s´appuie sur un institutionnel désigné et cadre juridique.	Oui /Non Exemples: Les principales caractéristiques du système sont définies dans des textes juridiques (couverture, l´ensemble des prestations, financement, provision, contrôle réglementaire et la gouvernance institutionnelle). Le cadre est approprié compte tenu du contexte de la CMB du pays (c´est-à-dire qu´il n´est pas trop restrictif, il considère les circonstances spéciales locales, et il n´ignore pas les parties importantes ou les acteurs dans le système). Le statut et les responsabilités de chaque différente institution de la CMB dans le système est clairement défini et transparent.
7. Des informations claires, les règles de diffusion et de transparence sont mises en place.	Oui / Non Exemples: Les lois sur la divulgation explicite existent dans la loi ou règlements de la loi. Les activités commerciales, la propriété et les situations financières sont régulièrement rendus public (c´est-à-dire les règles sont suivies). Les bénéficiaires ont accès aux informations financières des caisses maladie.
8.Les entités de la CMB ont un minimum d´exigences relatives à la protection de l´assuré.	Oui/Non Exemples: Les règles de protection du consommateur existent dans la loi, y compris l´information du consommateur, et les mécanismes indépendants de règlement des réclamations, les recours, les doléances et les conflits. L´assuré peut obtenir en temps utile, des informations complètes et pertinentes sur les changements des bénéfices ou la longueur de la couverture, etc. Les mécanismes de plainte du consommateur existent et sont pratiqués.

Source: Savedoff WD. Governing mandatory health insurance: concepts, framework, and cases. Gov Mandat Health Insur Learn Exp Wash DC World Bank. 2008;13-48. (Traduction libre)

**Tableau 4:** dimension supervision et régulation

Fonction	Indicateurs
9. Règles sur la conformité, l´application et les sanctions pour la supervision de la CMB sont clairement définies.	Oui /Non Exemples: Les règles de conformité et les sanctions sont définies dans textes juridiques. Des mesures correctives sont imposées, sur la base des critères objectifs qui sont publiquement divulgués. Capacité adéquate pour l´exécution de ces fonctions est prévue. Les cas de violation des règles et les actions subséquentes par le régulateur sont publiés.
10. Les règles de la gestion financière pour les entités de la CMB sont clairement définies et imposées.	Oui/Non Exemples: Les normes financières pour les entités de la CMB sont définies dans textes juridiques ou des règlements. Les règles financière d´autorisation / entrée sur le marché sont clairement définis (exigences du capital minimum). Les exigences en matière de la réserve en cours et de solvabilité sont définies. Le règlement des actifs et des placements financiers sont définis. Les règles d´audit (interne et externe) sont définies. Les règles relatives aux normes financières sont appliquées.
11. Le système de la CMB a des structures pour la supervision permanente et le monitoring en place.	Oui/ Non Exemples: Les règles non-financières pour l´obtention de la licence de rentrée sur le marché sont clairement définies. L´enregistrement des produits d´assurance est défini et réglementé. Inspections sur place adéquates et surveillance hors du site sont en place. Les règles de déclaration financière en cours sont définies et les informations fournies sont précises et opportunes. Des règles claires régissant la sortie / dissolution du marché sont en place.

Source: Savedoff WD. Governing mandatory health insurance: concepts, framework, and cases. Gov Mandat Health Insur Learn Exp Wash DC World Bank. 2008;13-48. (Traduction libre)

**Tableau 5:** dimension cohérence et stabilité

Fonction	Indicateurs
12. Les principales qualités du système de la CMB sont stables.	Oui/ Non Exemples: Les objectifs sont restés essentiellement les mêmes dans le passé récent. Les caractéristiques fondamentales du système de la CMB (par exemple, panier de prestations, les règles d´affiliation, les exigences de contribution, droits de protection de base pour l´assuré et les exigences institutionnelles de base pour les opérateurs) sont définies dans la loi. La loi est restée considérablement la même dans le passé récent (c´est-à-dire indépendamment des élections politiques ou crises économiques).

Source: Savedoff WD. Governing mandatory health insurance: concepts, framework, and cases. Gov Mandat Health Insur Learn Exp Wash DC World Bank. 2008;13-48. (Traduction libre)

## Résultats

**Description du système de gouvernance de la CMB au Maroc:** le modèle de gouvernance de la CMB après la sortie de la loi 65-00 est composé de plusieurs acteurs qui occupent un rôle dans le système comme le suivant: la chefferie du gouvernement: supervision du chantier de la CMB; la CNOPS: gestion du régime AMO public; la CNSS: gestion du régime AMO privé; l´ANAM: régulation de l´AMO et gestion des ressources financières du RAMED; le Ministère de la santé: met en place la stratégie de la couverture médicale du gouvernement et assure l´offre des soins publics; le ministère de l´intérieur: étude de l´éligibilité des bénéficiaires RAMED et distribution des cartes; l´ACAPS: contrôle financier des organismes gestionnaires (CNOPS et CNSS), ainsi que les mutuelles; le ministère du travail et de l'insertion professionnelle: tutelle des organismes gestionnaires (CNOPS et CNSS) et pilotage du système de la protection sociale.

**Analyse documentaire:** selon notre recherche, il y a une absence des articles scientifiques qui ont étudié la gouvernance et son impact sur le système de la CMB au Maroc. Nous avons analysé les publications émanant des autorités gouvernementales qui ont traité la gouvernance de la CMB, ainsi que les rapports des organismes internationaux qui ont travaillé en collaboration avec le gouvernement marocain pour des projets en relation avec la gouvernance de la CMB. La banque africaine du développement dans son rapport d´évaluation dans le cadre du Programme d´appui à la réforme de la couverture médicale - Phase 3 (PARCOUM III) a diagnostiqué la gouvernance de la CMB. Il a analysé le renforcement du comité interministériel chargé des orientations et décisions stratégiques de la réforme comme mesure dans le cadre d´un objectif de « Renforcer le mécanisme de pilotage et de gouvernance de la réforme » à moyen terme [4]. La coopération entre l´Organisation Mondiale de la Santé (OMS) et le ministère de la santé 2017-2021 prévoit un axe dédié à l´amélioration de la gouvernance du système de santé. Plusieurs actions qui sont prévues ont un retombé sur l´atteinte de la CSU et le renforcement de la gouvernance de la CMB [5]. La volonté d´amélioration de la gouvernance de la CMB au Maroc a été concrétisée aussi par le projet de partenariat avec l´union européen intitulé: « Renforcement de la gouvernance et du suivi de la couverture médicale de base au Maroc » [6].

Le Conseil Economique Social et Environnemental (CESE) dans son étude sur «la protection sociale: revue, bilan et renforcement des systèmes de sécurité et d´assistance sociale au Maroc», a mis l´accent sur les problèmes de gouvernance de la CMB [7]. Les résultats de l´analyse documentaire ont montré aussi l´existence d´un arsenal juridique important sur la CMB au Maroc dont on va détailler lors de la description des éléments relatifs au référentiel d´évaluation par la suite.

**Entretiens avec les acteurs de la CMB:** la majorité des informations collectées auprès des représentants figurent dans les textes juridiques et les rapports traitant la gouvernance de la CMB au Maroc que nous avons traité lors de l´analyse documentaire. L´apport des entretiens était principalement de donner des commentaires à intégrer dans l´évaluation par dimension et fonction du référentiel utilisé. Les interlocuteurs ont jugé que le système de gouvernance de la CMB nécessite une amélioration et qu´ils ont participé à des réflexions d´évaluation du modèle existant dans le cadre des projets de partenariats. En ce sens, la majorité a la conviction que l´amélioration de la gouvernance aura un impact majeur sur la performance du système de la CMB via le perfectionnement des services offerts, l´optimisation des ressources et le renforcement la satisfaction et la confiance du citoyen. L´ensemble des interrogés a déclaré avoir ignoré l´existence d´un référentiel d´évaluation de la gouvernance de la CMB. In fine, les représentants des acteurs de la CMB interrogés lors des entretiens ont montré que la synergie entre les acteurs de la CMB doit être renforcée et que chaque acteur devra participer à la prise en considération de l´intérêt final qui est la satisfaction du citoyen.

### Evaluation du modèle de gouvernance de la CMB au Maroc

#### Dimension 1: cohérence de la structure de prise de décision

Les taux de cotisation, le panier de soins et les taux de remboursement pour les deux régimes Assurance Maladie Obligatoire et Régime d´Assistance Médicale sont mentionnés dans les textes juridiques. Les décisions concernant la stratégie de la CMB sont prises au niveau de la commission interministérielle au niveau de la chefferie du gouvernement. Les décisions qui concernent l´équilibre financier du régime AMO sont prises au niveau du conseil d´administration de l´ANAM dans sa session AMO. Les décisions stratégiques concernant le RAMED sont prises lors de la commission interministérielle, le conseil d´administration de l´ANAM dans sa session RAMED discute les problèmes de gestion opérationnelle. L´ANAM ne dispose pas d´un accès total aux données des organismes gestionnaires (CNOPS et CNSS), chose qui ne lui permet pas d´assumer ses responsabilités et de prendre des décisions solides. Concernant le RAMED, les responsabilités des organismes selon la loi 65-00 [8] ne sont pas respectées, car l´ANAM ne gère pas les ressources du RAMED. L´ANAM suit l´équilibre budgétaire de l´AMO par l´élaboration d´un rapport annuel qui est présenté lors du conseil d´administration. Ce rapport détaille les recettes et les dépenses du régime. Les organismes gestionnaires de l´AMO suivent et analysent l´évolution de leurs recettes et dépenses et présentent un rapport annuel lors des conseils d´administration. La CNSS dispose d´un Comité «Audit et Risques» qui a un rôle consultatif, notamment en ce qui concerne l´établissement des comptes, les missions des auditeurs et commissaires aux comptes, le dispositif de contrôle interne et les risques. Selon le rapport du CESE [7]: «*Les attributions des conseils d´administrations des organismes gestionnaires des régimes de prévoyance sociale varient aussi, et restent généralement limités, en matière de délibération et de contrôle sur les opérations de gestion de la trésorerie et de placement des réserves financières, en matière de contrôle des risques, d´audit interne, et de nomination et de rémunération des dirigeants exécutifs ainsi qu´en matière de statut et de gestion des ressources humaines des organismes en question*».

#### Dimension 2: intervention des parties prenantes

Au niveau stratégique de la CMB et dans la commission interministérielle de la CMB, tous les acteurs de la CMB sont représentés à savoir: l´ANAM, la CNOPS, la CNSS, le Ministère de la santé, le Ministère des finances et Ministère de l´emploi. AMO: le conseil d´administration de l´AMO est organisé à l´ANAM et présidé par le chef du gouvernement ou l´autorité gouvernementale déléguée par lui à cet effet qui est souvent le Ministre de la santé. Sa composition contient plusieurs représentants des entités gouvernementales et détaillée dans le Décret n° 2-03-402 [9].

Les décisions du conseil sont prises à la majorité des voix des membres présents. En cas de partage égal des voix, celle du président est prépondérante. Le représentant de l´ANAM voit que: «*la composition du conseil d´administration doit être revue parce qu´elle est similaire aux conseils d´administration des organismes gestionnaires. Selon lui, un organe de régulation doit avoir dans son conseil d´administration les décideurs de l´assurance maladie au Maroc*». Le conseil d´administration de la CNSS est composé de 24 membres titulaires, nommés par décret pour une période de 3 ans, dont: 8 représentants de l´état; 8 représentants des employeurs; 8 représentants des travailleurs. Le conseil d´administration est présidé par le chef du gouvernement ou par l´autorité gouvernementale déléguée par lui à cet effet qui est le Ministre de l´emploi. Les décisions du conseil sont prises à la majorité des voix des membres présents. En cas de partage égal des voix, celle du président est prépondérante. La CNOPS a deux conseils d´administration: un conseil d'administration pour le régime des salariés publics composé, pour moitié, des représentants de l'Etat et pour moitié des présidents des sociétés mutualistes la composant ainsi que des représentants des centrales syndicales les plus représentatives; un conseil d´administration pour le régime des étudiants doit se tenir séparément de la gestion de toute autre couverture médicale que la CNOPS assure. Il est composé de son Président et dix membres titulaires [10].

**RAMED:** le conseil d´administration du RAMED est organisé à l´ANAM et présidé par le chef du gouvernement ou l´autorité gouvernementale déléguée par lui à cet effet. Sa composition est détaillée dans le Décret n° 2-03-402 [9].

#### Dimension 3: transparence et accès à l´information

L´article 31 de la constitution marocaine de 2011 mentionne pour la première fois au Maroc le droit à la couverture médicale [11]. La loi 65-00 a annoncé les objectifs, les structures, les règles de gestion, les taux de cotisation et de remboursement... Tous les documents juridiques (Lois, Décrets, Arrêtés) en relation avec la CMB sont accessibles pour le grand public via le site web de l´ANAM [12]. L´article 1 de la loi 65-00 mentionne qu´il est institué un système de couverture médicale de base comprenant l'Assurance Maladie Obligatoire (AMO) et le Régime d'Assistance Médicale (RAMED). L´assurance maladie obligatoire de base est fondée sur le principe contributif et sur celui de la mutualisation des risques. Le régime d'assistance médicale est fondé sur le principe de la solidarité nationale au profit de la population démunie. Les personnes assurées dans ce cadre et les bénéficiaires doivent être couverts sans aucune discrimination due à l'âge, au sexe, à la nature de l'activité, au niveau et à la nature de leur revenu, à leurs antécédents pathologiques ou à leurs zones de résidence. La tarification nationale de référence est basée sur les conventions signées entre les organismes gestionnaires et les professionnels de santé et elle a été publiée dans des arrêtés entre les années 2006 et 2007 [13]. Il n´y a pas de cadre institutionnel de gestion du RAMED car le modèle primaire proposé dans la loi 65-00 n´as pas été appliqué et l´ANAM ne gère que la production des cartes RAMED et non pas ses ressources financières. Les sites web des organismes gestionnaires publient les rapports annuels d´activité incluant toutes les statistiques sur la population couverte ainsi que les informations financières. Les deux sites web permettent aussi aux assurés de suivre le statut des déposés avec la possibilité de télécharger une application dédiée au Smartphone. Les organismes gestionnaires disposent d´une procédure pour réceptionner la réclamation des assurés auprès des établissements et professionnels de soins. En cas de litige, l´assuré peut déposer une réclamation au niveau de l´ANAM qui a une mission d´arbitrage, et qui doit régler son problème ou lui répondre officiellement par lettre recommandée. L´ANAM publie des chiffres sur les réclamations reçues sur le rapport d´activité annuel. Le bénéficiaire du RAMED a le droit aussi de déposer sa plainte au niveau des services de ministère de l´intérieur en cas de problème d´éligibilité, et à l´ANAM pour tout litige lié au processus d´octroi de la carte ou à l´offre de soins dans l´hôpital public.

#### Dimension 4: supervision et régulation

Dans le titre premier du livre IV de la loi 65-00 intitulé: contentieux, recours, sanctions et subrogation, il est mentionné les amendes suivantes [10]: les amendes des employeurs dans le cas du non-paiement des cotisations de leurs salariés, les amendes des prestataires de soins qui se rendent coupables de fraude ou de fausse déclaration ou pour le refus du contrôle médical et les amendes des organismes gestionnaires en cas de la non communication des statistiques annuelles à l´ANAM. L´ANAM peut déconventionner tout praticien ou établissement des soins qui ne respectent pas la tarification en vigueur, mais cette règle est rarement appliquée. Pour le RAMED, il n´y a pas de textes juridiques qui déterminent les sanctions liées à la procédure de l´octroi de la carte ou aux conditions de l´offre de soins offerte aux Ramédistes. L´article 52 de la loi 65-00 [10] mentionne que les comptes et opérations des organismes gestionnaires sont soumis annuellement à un audit comptable et financier externe diligenté par le conseil d'administration de l'organisme concerné. Les règles des placements des organismes gestionnaires et de l´ANAM sont unifiées avec celles des autres établissements publics au niveau de la caisse de dépôt et de gestion qui est désignée comme organisme dépositaire, en application des dispositions de l´article 5 du décret n° 2-05-740 du 11 joumada II 1426 (18 juillet 2005) susvisé. Pour le RAMED il n´y a pas de textes juridiques qui cadrent sa gestion financière dont l´absence d´un organisme gestionnaire de ce régime. L´ANAM est désignée comme étant le régulateur de l´AMO depuis sa création et elle est chargée du suivi de l´équilibre budgétaire, le monitoring financier du régime ainsi que la mise en œuvre des outils de régulation. Selon le représentant de l´ANAM: « *la tutelle de l´ANAM doit être liée à la chefferie du gouvernement, car le ministère de la santé en tant que producteur de soins et acteur principal dans la CMB ne doit pas être tutelle d´une agence de régulation comme l´ANAM*». Il y a aussi L´ACAPS, qui est un organisme doté de la personnalité morale et de l´autonomie financière. Elle est chargée du contrôle financier du secteur des assurances, des organismes gestionnaires de l´assurance maladie obligatoire, de la retraite et des organismes de droit privé gérant les opérations de retraite fonctionnant par répartition ou par répartition et capitalisation. Une Commission Interministérielle de Pilotage et la Commission Technique Interministérielle ont été instituées en 2013 par une circulaire [14], en vue de permettre aux différents acteurs de travailler de façon convergente afin de renforcer l´accès aux soins de qualité, la généralisation de la couverture en garantissant la pérennité des régimes. La circulaire a institué le cadre global de l´élaboration de la stratégie de réforme de la CMB en tant que politique publique. Cette commission a tenu plusieurs réunions techniques et de pilotage depuis sa création et plus spécialement pour suivre le lancement du régime des indépendants de l´AMO. Toutefois, en 30 mars 2018, le chef du gouvernement a lancé une nouvelle circulaire [15], instituant une commission pour la gouvernance de la protection sociale (technique et de pilotage), qui remplace la première circulaire dans une vision plus globale et pour un objectif de converger les efforts déployés pour fournir au citoyen marocain un service de protection sociale intégré et complet. Cette commission a donné lieu à quatre cellules de travail notamment: la gouvernance et la convergence des programmes de la protection sociale, la couverture médicale de base (sous responsabilité de ministère de la santé), l´assistance sociale et l´approche de ciblage. Selon le représentant du ministère de l´emploi: « *la cellule de la couverture médicale de base ne doit pas être sous responsabilité du Ministère de la santé vu son statut de l´offreur de soins public, elle doit être supervisée par une entité gouvernementale qui ne figure pas parmi les parties prenantes de la CMB fin qu´elle statue sur les vrais problèmes et prenne les décisions les plus pertinentes*».

#### Dimension 5: cohérence et stabilité

La constitution de 2011 a nouvellement introduit le droit à la couverture médicale, chose qui a donné plus de consistance au système de la CMB lancé en 2002 après la sortie de la loi 65-00. Le régime AMO a gardé une stabilité en matière des règles d´affiliation avec l´introduction du contrôle de la double immatriculation comme nouvelle mission de l´ANAM. La tarification nationale de référence n´a pas été changée depuis les conventions signées en 2006-2007, or, l´écart avec les prix du marché et cette tarification devient de plus en plus importante. Le changement était pour le mode de gestion financière du RAMED qui n´a pas reflété les dispositions juridiques de la loi 65-00 qui donne à l´ANAM cette mission, et nous vivons aujourd´hui un réel problème face à cette ambiguïté. En matière de supervision stratégique, la CMB a été influencé par la volonté politique reflétée par la généralisation du RAMED en 2012 pour toutes les régions du Maroc et la constitution de la commission interministérielle de la CMB par la chefferie du gouvernement en 2013 pour le suivi du chantier en tant que politique publique. Le chef du gouvernement a annoncé la création d´une commission interministérielle de pilotage de la réforme du système de protection sociale dans une circulaire émise le 30 mars 2018 et adressée à 12 ministères. Ce document a mis l´accent sur la nécessité d´accélérer pour la généralisation de la couverture médicale et résoudre les problèmes de financement du RAMED. Ce qui met en évidence la volonté politique du gouvernement pour assurer la continuité de travail accompli dans le chantier de la CMB et d´améliorer les conditions actuelles du service fourni au citoyen marocain.

## Discussion

Comme il est prévu dans les textes réglementaires, la CMB lors du lancement de la loi 65-00 avait son modèle de gouvernance pour les deux pôles AMO et RAMED. Aujourd´hui, ce modèle fait face à plusieurs dysfonctionnements, nouveaux défis et des opportunités à saisir pour assurer sa pérennité. Le système de la CMB a été lancé pour fournir à la population marocaine une couverture obligatoire contre le risque maladie, et il s´inscrit dans les objectifs lancés par l´OMS sur la couverture sanitaire universelle. Il s´agit des Résolutions de l´Organisation Mondiale de la Santé (OMS) en 2005 et 2011 (WHA58.33 et WHA64.9) [2] liées à la Couverture médicale universelle. Partant de cette base, l´objet de cette étude était d´analyser le modèle de gouvernance de la CMB afin de proposer les pistes d´amélioration. Il ressort de cette étude les éléments importants que nous détaillons dans les lignes suivantes.

### Pour la gouvernance globale de la CMB

Le Maroc s´est penché sur la question de la Couverture médicale depuis un bon moment depuis la période du protectorat avec la création des mutuelles. Avant la loi 65-00, nous ne pouvons pas parler d´un modèle de gouvernance car l´adhésion était facultative avec l´absence d´une stratégie globale du gouvernement pour ce chantier. La version de 2002 du modèle de gouvernance suite à plusieurs années de réflexion était le compromis entre le gouvernement et les syndicats en prenant en considération les institutions déjà en place comme les mutuelles, la CNOPS et la CNSS. La commission interministérielle a joué un rôle très important depuis sa création en 2013 pour concrétiser la volonté du gouvernement pour accélérer l´atteinte de la CSU. Cependant, cette commission devait voir le jour depuis le lancement de la loi 65-00, car durant toute cette période il n´y avait pas une supervision régulière pour ce chantier par le gouvernement. La commission interministérielle de mars 2018 de la protection sociale constitue un point positif dans la gouvernance de la CMB, du fait qu´il va veiller à la complémentarité entre la CMB et les autres régimes des risques sociaux, or la supervision de la cellule de la CMB au sein de cette commission par le Ministère de la santé doit être changée vue le statut de l´offreur de soins que joue toujours cette ministère. La problématique de la gouvernance de la CMB a été analysée dans plusieurs rapports institutionnels et dans le cadre des projets de partenariat comme nous avons mentionné. Il est à noter que malgré toute la réflexion menée autour de cette thématique, aucune action gouvernementale n´est vu le jour jusqu´à ce jour. Il s´est avéré aussi, et malgré la création de la commission de la protection sociale par le chef du gouvernement, que nous n´avons pas encore tranché sur un positionnement de la CMB stratégiquement parlant en tant que chantier public. D´un point de vue, il y a des acteurs qu´ils l´attachent au ministère de la santé et à sa stratégie sectorielle. D´autres acteurs, estiment que nous devons avoir un programme national de la sécurité sociale incluant la couverture du risque maladie. Il y a aussi un troisième avis qui place la CMB comme politique publique autonome qui nécessite une mobilisation de tous les acteurs pour atteindre la CSU comme le recommande l´OMS. Il est à noter aussi que nous n´avons pas trouvé dans la mise en œuvre de la CMB une complémentarité entre les deux régimes AMO et RAMED, ce qui met en question le principe de la solidarité sur lequel se base le système. La difficulté se résume dans le fait de construire un modèle de gouvernance en capitalisant sur l´existant (structures, organismes, textes juridiques) tout en changeant les paramètres qui nuisent aux principes internationaux de la bonne gouvernance.

### Pour la gouvernance de l´AMO

L´ANAM en tant qu´une nouvelle agence de régulation, a pu concrétiser pas mal de dispositions de la loi 65-00 telles que: les conventions entre les organismes gestionnaires et les professionnels de santé, le suivi de l´équilibre financier, la normalisation des feuilles de soins etc. Cependant, son rôle est devenu de plus en plus limité face l´absence d´une volonté politique de sa tutelle pour lui donner le pouvoir qu´il faut pour assurer ses missions. Le ministère de la santé en tant que tutelle de l´ANAM, préside ses conseils d´administration au moment où les textes précisent qu´il y a lieu de confier cette présidence à la chefferie du gouvernement. De même, la représentation au sein des conseils d´administration des EEP souffre de l´absence d´un dispositif précis en la matière. Le ministère de la santé chapote réellement le chantier de la CMB qui constitue un axe stratégique dans sa stratégie sectorielle. La réalité montre qu´il y a des chevauchements entre le rôle du ministère et celui de l´ANAM, ce qui met en cause son rôle du régulateur qui doit être à distance de tous les acteurs de la CMB, notamment le ministère de la santé, qui est le responsable de l´offre de soins public. L´ANAM ne peut pas prendre des décisions stratégiques et proposer des changements dans le système de la CMB car elle ne dispose pas d´un accès total aux données des organismes gestionnaires (CNOPS et CNSS) et dans l´absence d´un système d´information intégré. Le conseil d´administration de l´ANAM ne peut pas trancher sur des questions stratégiques de l´AMO, ces points sont renvoyés à la commission interministérielle au niveau de la chefferie du gouvernement. Dans quelques cas, les orientations stratégiques sont arrêtées en interne, le conseil d´administration se contente de valider les choix du management. Depuis 2006, l´ANAM n´a pas pu renouveler les conventions entre les professionnels de santé et les organismes gestionnaires, ce qui met en cause le pouvoir de cet organisme à jouer le rôle de médiateur pour ce dossier qui demande un appui politique et stratégique du gouvernement. 12 ans après la création de l´ANAM, le modèle marocain pour la régulation de la CMB par une agence indépendante a montré ses limites, car l´agence est confrontée dans sa mission à des organismes gestionnaires qui ont plus de poids institutionnel que celui dont elle dispose, et à un groupement des professionnels de santé cadré par les prix du marché. Autrement dit, l´ANAM n´a pas pu imposer sa vision et réussir la mission de la régulation comme elle est détaillée dans la loi. Le rôle d´arbitrage de l´ANAM ne donne pas une sécurité pour le citoyen face à un problème avec un établissement de soins, car elle ne dispose pas de pouvoir de sanction en cas de surfacturation ou de camouflage de l´information. Le mode de travail de la CNOPS et les mutuelles posent beaucoup de problèmes actuellement car il n´a pas été revu depuis la création des mutuelles, et bien que l´article 44 de la loi 65-00 interdisent pour les organismes gestionnaires d´offrir les soins, les mutuelles fournissent toujours les soins ambulatoires pour les assurés AMO. Le régime des étudiants a fait rentrer des nouveaux acteurs dans la gouvernance de l´AMO comme l´Office de la Formation Professionnelle et de la Formation au Travail (OFPPT) et l´Office Nationale des Œuvres Universitaires Sociales et Culturelles (ONOUSC). Des nouvelles attributions sont affectées à l´ANAM notamment la mise à jour du référentiel des établissements de formation. Toutefois, il y a plusieurs problèmes d´ouverture de droit dans le système d´information vu le manque de coordination entre les acteurs de ce régime pour actualiser la base de données en vue du contrôle de la double immatriculation.

Il n´y a pas une visibilité sur la stratégie du gouvernement pour intégrer à l´AMO la population couverte par les organismes ayant des caisses internes comme l´indique l´article 114 de la loi 65-00. Actuellement l´Office National des Chemins de Fer (ONCF) est le seul organisme qui a intégré l´AMO public. Le contrôle des dépenses se fait au niveau des conseils d´administration. Malgré que les organismes gestionnaires offrent un service de suivi des dossiers de remboursement en ligne, l´information n´est pas disponible lors d´un retard de traitement d´un dossier qui ne doit pas dépasser trois semaines selon la réglementation.

Il faut signaler également l´absence d´un dispositif réglementaire clair et bien défini qui précise les conditions de nomination des représentants de l´Etat au sein des conseils d´administration, le manque d´efficacité de certains organes délibérants qui souffrent de carences institutionnelles et organisationnelles, ce qui accentue les limites de leurs capacités décisionnelles. Le caractère pléthorique des membres des conseils de certains EEP ne favorise pas les conditions d´un débat de qualité. Les représentants des acteurs agissent souvent en porte-parole de leurs administrations respectives en reléguant au second plan leur rôle de défendre et de préserver l´intérêt du citoyen. Les administrateurs désignés pour participer aux conseils d´administration discutant des points fortement techniques, n´ont pas toujours les compétences, ni les qualifications professionnelles pour assumer la fonction du représentant.

Le budget qui est l´acte essentiel dans la vie des établissements publics à travers la traduction de l´effort d´investissement envisagé, est discuté au préalable avec le ministère de l´économie et des finances, le conseil d´administration ne fait qu´entériner a postériori. En conséquence, le conseil se dessaisit, de fait, de sa fonction principale, à savoir l´orientation stratégique. La société civile qui défend le droit à la santé et les droits du consommateur dans le domaine de la santé n´est pas présentée dans les conseils d´administration.

### Pour la gouvernance du RAMED

La gouvernance du RAMED a plusieurs dysfonctionnements qui influencent la stabilité du régime. Le fait de priver l´ANAM de sa mission de gestion des ressources financières du RAMED a créé un déséquilibre, et jusqu´à ce jour, le gouvernement n´a pas trouvé une solution optimale pour ce problème, ce qui met en question sa viabilité dans l´avenir. Le conseil d´administration du RAMED ne dispose pas d´une représentativité des bénéficiaires, chose qui s´oppose au principe de l´intervention de toutes les parties prenantes pour la prise de décision. La procédure du contrôle de la double immatriculation effectuée par l´ANAM, pose plusieurs problèmes, car il n´y a pas de système d´information actualisé au temps réel entre les acteurs du RAMED, ce qui retarde l´ouverture de droit et la production de la carte du bénéficiaire souvent en situation d´urgence médicale. Les Ramedistes sont confrontés au manque d´information pour leur droit à la couverture médicale, car la plupart sont des personnes analphabètes. En plus, le bénéficiaire de ce régime ne peut accéder qu´à l´offre de soins dans les hôpitaux publics, et lorsqu´il y a un problème d´accès aux soins (anomalie d´un scanner, non disponibilité du médecin, rendez-vous à une date lointaine pour la consultation ou pour l´acte médical etc.), il est privé de ce droit qui est un besoin vital. Le RAMED souffrent des problèmes relatifs à la lenteur dans le processus de production de carte d´un côté, et de l´autre côté, aux insuffisances de l´offre de soins dans l´hôpital public. Le RAMED à l´opposé de l´AMO, n´a pas d´organisme gestionnaire dédié, sa gestion est divisée entre plusieurs organismes. La gestion des ressources est assurée par le Ministère de la santé et le Ministère d´Economie et des Finances, ce qui entrave la séparation entre la gestion des ressources et la prestation de soins. Ce mécanisme nuit aux principes de la bonne gouvernance. S´ajoute à cela, l´absence d´un système de comptabilité propre au RAMED dans les hôpitaux publics, ce qui cause des problèmes de traçabilité et de suivi des ressources dépensées relatives au RAMED.

**Limitations:** la limite majeure de l´étude consiste à la rareté des référentiels spécialisés dans la gouvernance de la CMB, vu que le seul que nous avons trouvé dans notre recherche est celui de la banque mondiale. La méthodologie de l´étude nous a montré que le concept de la gouvernance est difficile à cerner que chaque partie prenante en a sa propre compréhension. Ce qui nous a incité lors des entretiens à faire un effort pour unifier le concept avant de discuter son application dans le domaine de la CMB.

## Conclusion

La couverture médicale de base (CMB) est un chantier de première nécessité pour la population d´un pays, il participe directement dans l´amélioration des indicateurs sociaux et constitue un support majeur pour la cohésion sociale. Le Maroc, a marqué des grands pas en matière de CMB. L´objectif aujourd´hui est de surmonter les défis actuels et atteindre la CSU qui reste un chantier plus global qui aura un impact sur tout le système de santé. Notre étude s´est focalisée sur le volet gouvernance afin de le rendre compréhensible et lever son ambiguïté, d´une part. De l´autre part, l´étude a permis de mesurer la gouvernance et la dissocier du management public de la CMB au Maroc. Nous avons pu transposer le modèle proposé par la banque mondiale en cinq dimensions sur le cas marocain afin de diagnostiquer les problèmes et proposer des solutions optimales. Il s´avère indispensable de mentionner que la réussite du système de la CMB au Maroc nécessite une vraie volonté politique pour défendre l´intérêt du citoyen en améliorant la qualité du service offert dans le régime AMO et la mise à niveau de l´offre de soins publics pour le RAMED. Une adhésion de toutes les parties prenantes dans la prise de décision concernant les orientations stratégiques et les éventuelles reformes créera un climat de confiance et va faciliter l´application des mesures fixées. In fine, nous pouvons ajouter que, sans un système de valeurs partagées entre les acteurs du système de santé (Etat, professionnels de santé, patient), la CMB ne pourra pas atteindre des objectifs. Une gouvernance, sans prise de décision éthique qui respecte les valeurs de la société dans une optique d´améliorer la vie du citoyen, reste dépourvue de son sens.

### Etat des connaissances sur le sujet

- Existence d´un référentiel d´évaluation des modèles de la gouvernance de la CMB composé de 5 dimensions en 2008 par Savedoff et Gottret dans le cadre de l´étude de la banque mondiale «Governing Mandatory Health Insurance: Learning from Experience»;
- Plusieurs réflexions ont été lancées au Maroc pour améliorer la gouvernance du système existant de la CMB par des institutions publiques.

### Contribution de notre étude à la connaissance

- Evaluation du modèle de gouvernance de la CMB au Maroc selon le référentiel de la banque mondiale.
